# Residual, Enrichment and Health Risk Assessment of Hexachlorocyclohexane and Dichlorodiphenyltrichloroethane in Muscle of Cultured Common Carp

**DOI:** 10.3390/foods14020223

**Published:** 2025-01-13

**Authors:** Li Huang, Lei Gao, Song Wu, Peng Wang, Zhongxiang Chen, Qirui Hao, Dongli Qin, Xiaoli Huang

**Affiliations:** 1 Heilongjiang River Fisheries Research Institute, Chinese Academy of Fishery Sciences, Harbin 150070, China; huangli@hrfri.ac.cn (L.H.); gaolei@hrfri.ac.cn (L.G.); wusong@hrfri.ac.cn (S.W.); wangpeng@hrfri.ac.cn (P.W.); chenzx@hrfri.ac.cn (Z.C.); haoqirui@hrfri.ac.cn (Q.H.); 2 Inspection and Testing Center for Fishery Environment and Aquatic Products (Harbin), Ministry of Agriculture and Rural Affairs, Harbin 150070, China; 3 Heilongjiang River Basin Fishery Ecological Environment Monitoring Center, Ministry of Agriculture and Rural Affairs, Harbin 150070, China

**Keywords:** hexachlorocyclohexane, dichlorodiphenyltrichloroethane, enrichment, common carp, health risk assessment

## Abstract

Common carp (*Cyprinus carpio*) is extensively cultured and widely consumed in Heilongjiang Province, China. Due to the proximity of freshwater ponds to agricultural cultivated areas, these aquatic systems are inevitably influenced by the historical application of organochlorine pesticides (OCPs), due to their prolonged half-life and resistance to degradation. Gas chromatography-tandem mass spectrometry (GC-MS/MS) was utilized to quantify the levels of hexachlorocyclohexane (HCHs) and dichlorodiphenyltrichloroethane (DDTs) in the muscle tissue of cultured common carp. This study examined the enrichment characteristics of HCHs and DDTs in fish muscle, as well as their correlation with sediment and associated risk assessments. The results showed that the residual levels of HCHs and DDTs in fish muscle ranged from 0.387 μg·kg^−1^ to 3.418 μg·kg^−1^ and from 0.114 μg·kg^−1^ to 0.420 μg·kg^−1^, respectively. They were all below the maximum residue limits specified in GB 2763-2021 (HCHs: 100 μg·kg^−1^, DDTs: 500 μg·kg^−1^). The concentrations of HCHs and DDTs in sediment were found to be lower than those in muscle tissue. Notably, the concentrations of HCHs were higher than those of DDTs in both muscle and sediment. Among the HCHs, γ-HCH was the predominant residual substance, contributing a significant proportion of 42.16% to 86.47%. No significant residues of DDT derivatives were detected. A significant correlation was observed between the concentrations of HCHs and DDTs in the muscle tissue and those present in the sediment (*p* < 0.01). The health risk assessment indicated that both the carcinogenic and non-carcinogenic risks associated with OCPs from common carp muscle and sediment were within acceptable limits. Therefore, it was recommended to regulate fish consumption during the breeding period.

## 1. Introduction

Organochlorine pesticides (OCPs), which are chlorinated aromatic hydrocarbon derivatives of high toxicity, long half-lives, and challenging degradation [1]. They possess the attributes of bioaccumulation and long-distance atmospheric migration [2]. Despite China’s discontinuation of production, sale, and application of these pesticides in 1980 [3,4], they can migrate and undergo transformation in various environmental media such as soil [5], the atmosphere [6], and water [7], ultimately accumulating and amplifying within organisms through the food chain, thereby posing a threat to the ecological environment and human health [8].

Heilongjiang Province is renowned for its fishery resources and extensive cultivated land resources [9]. Statistical data from 2023 indicates that Heilongjiang Province ranked second in the nation for common carp (*Cyprinus carpio*) production, contributing 8.68% to the total national output of cultured common carp. Additionally, common carp cultivation constituted 34.4% of the freshwater aquaculture output in Heilongjiang Province [10]. However, the freshwater fish were cultivated in soil-based ponds situated in proximity to farmland. Heilongjiang Province has the largest cultivated land area in the country, where OCPs have been historically utilized. Pesticides, being the foremost method employed to combat crop diseases and pests [11], have been found to have significantly detrimental effects on human health and the ecological environment [12]. A multitude of studies conducted by researchers have yielded specific findings regarding the residual quantity, origin, and assessment of health risks associated with OCPs in various types of soil, such as urban soil [13,14], farmland soil [15,16], surface soil [17] and watershed soil [18]. However, there is a dearth of literature on the residual characteristics of OCPs in the sediment of soil-based ponds located in close proximity to cultivated land. The soil in neighboring cultivated land could contaminate the pond sediment through surface runoff and other means. The residues of hexachlorocyclohexane (HCHs) and dichlorodiphenyltrichloroethane (DDTs) in the soil might be transported into water bodies through rainfall.

Is there a potential for the migration and accumulation of HCHs and DDTs residues in pond bottom mud and aquaculture, ultimately affecting the cultured fish? Previous studies have detected the presence of HCHs and DDTs residues in fish from various regions in China, including Yunnan Province, Liaodong Peninsula, Shanxi Province, Fujian Province and coastal areas. However, there are few studies on OCPs in cultured common carp in soil ponds in Heilongjiang Province. The accumulation of HCHs and DDTs through the food chain could had adverse effects on quality and safety of aquatic products, posing a potential threat to human health. Heilongjiang Province, is the main production area of common carp culture and has a high levels of carp consumption; therefore, conducting safety and risk assessments of farmed fish is of significant importance in Heilongjiang Province.

This study aimed to investigate the residual levels of HCHs and DDTs in the muscle of cultured common carp cultured in freshwater ponds. Additionally, the composition characteristics of these contaminants were examined, and a correlation analysis with sediment was conducted throughout the culture period. The objective was to explore the potential health risks associated with consuming edible common carp and to provide a scientific basis and reference for ensuring the quality and safety of aquatic products.

## 2. Materials and Methods

### 2.1. Sample Collection

#### 2.1.1. Collection of Common Carp

Cultured common carp muscle samples: The experimental samples were randomly obtained from the breeding pond located in Bin County, Harbin City, in China (127°05′08″ E, 46°03′22″ N), a pond surrounded by farmland. Three common carp back muscles were mixed together as one sample. A total of 95 common carp samples were collected from April to September; the samples measured between 10.30 and 34.79 cm in length and weighed between 105.6 and 925 g. The specimens of common carp collected in each batch were maintained in cold storage. Upon arrival at the laboratory, the muscle tissue of cultured common carp was extracted and homogenized to produce surimi, which was then stored at −20 °C until chemical analysis was conducted within a predetermined timeframe.

#### 2.1.2. Collection of Sediment

A total of 95 samples of sediment were collected according to the requirements of the standard “Technical specification for soil environmental monitoring (HJ/T 166-2004 [19])”. Air-dried sludge was sieved through a 100-mesh screen and stored in a drying apparatus. Chemical analyses were performed within the specified timeframe.

### 2.2. Experimental Reagents and Instruments, Chemicals and Materials

HCHs (containing α-HCH, β-HCH, γ-HCH, δ-HCH) and DDTs (containing *p*,*p*′-DDD, *p*,*p*′-DDE, *o*,*p*′-DDT, *p*,*p*′-DDT) were selected in this study; all had >98% purity at 200 mg·L^−1^ in 1.2 mL in cyclohexane; the internal standard heptachlor epoxide had >98% purity at 100 mg·L^−1^ in 1.2 mL in cyclohexane. All were purchased from Anpel laboratory technologies (Shanghai) Inc. (Shanghai, China). Cyclohexane was used to prepare the individual stock standards as well as the isotopically labelled internal standards. The working standard mixes, 1.0 mg·L^−1^ (HCHs and DDTs) and 0.1 mg·L^−1^ (heptachlor epoxide), were also prepared by spiking each of the dissolved individual stock solutions with cyclohexane. Following that, cyclohexane was used to dilute the mixed working standard to the concentration required. All the standards were stored at −20 °C. All organic solvents (dichloromethane, n-hexane, cyclohexane, ethyl acetate, acetonitrile) (HPLC grade) and sodium sulfate and sodium chloride (analytical reagent) were purchased from Anpel laboratory technologies (Shanghai) Inc. (Shanghai, China). Ultrapure water was obtained using a Milli-Q water filtering device. Anpel provided organic needle filters (0.22 μm). Bio-Bead S-X3 gel was purchased from J2 Scientific (Columbia, MO, USA). A gas chromatography-tandem mass spectrometry (GC-MS/MS) (7890B~7000C) instrument was purchased from Agilent (Santa Clara, CA, USA).

### 2.3. Extraction and Purification of HCHs and DDTs

#### 2.3.1. Extraction of HCHs and DDTs in Common Carp

We accurately measured 5.0 g of common carp muscle tissue into a 50 mL centrifuge tube and then added 0.1 mL of internal standard solution (0.1 mg·L^−1^), 3 mL of ultrapure water and 20 mL of acetonitrile. The mixture was swirled and mixed well before undergoing ultrasonic extraction for 30 min. Then, 2.0 g sodium chloride and 8.0 g of anhydrous sodium sulfate were added, followed by centrifugation at 7000 r·min^−1^ for 5 min. The resulting supernatant was then transferred to a concentration tube. The extraction process was iterated using 20 mL of acetonitrile, and the resulting extracts were combined and concentrated to dryness utilizing a multi-sample quantitative concentration/parallel evaporation apparatus. The concentrated solution was subsequently reconstituted in a mixture of cyclohexane and ethyl acetate (V/V = 1 + 1), and filtered through a 0.22 μm organic phase filter to achieve a final volume of 10 mL for purification [20].

#### 2.3.2. Extraction of HCHs and DDTs in Sediment

A sediment sample weighing 5.0 g was placed into the ASE extraction tank of a fast solvent extractor, followed by the addition of 0.1 mL of internal standard solution (0.1 mg·L^−1^) and 50 mL of a mixed solvent of n-hexane-dichloromethane (V/V = 1 + 1). The ASE conditions included a temperature of 100 °C, pressure of 10.34 MPa, static extraction for 5 min, and three cycles. The resulting liquid was concentrated to near-dryness using a multi-sample quantitative concentration/parallel evaporation apparatus, redissolved in cyclohexane-ethyl acetate (V/V = 1 + 1), and filtered by a 0.22 μm organic phase filter to a final volume of 10 mL for purification.

#### 2.3.3. Purification of HCHs and DDTs

The Bio-Bead S-X3 gel was used as column packing material, with a mobile phase consisting of cyclohexane-ethyl acetate (V/V = 1 + 1). The flow rate of the column was maintained at 4.7 mL·min^−1^, with a sample volume of 5 mL for quantitative analysis. Following purification via Accelerated Solvent Extraction (ASE), the effluent was collected between 8.0 and 18.5 min, concentrated online, and redissolved in 1.0 mL of n-hexane before injection of 1 μL into GC-MS/MS [20,21] using unsplit stream sampling. According to Formula (1),

(1) $$X=\frac{(C-C_{0})\times V}{m}$$

X is the content of HCHs or DDTs in the sample (μg·kg^−1^), C is the mass concentration of HCHs or DDTs in the sample liquid (μg·L^−1^), C_0_ is the mass concentration of HCHs or DDTs in the blank sample liquid (μg·L^−1^), V is the constant volume (mL), and m is the sample quantity (g).

Bioconcentration factors (BCFs) were used to evaluate the enrichment ability of HCHs and DDTs in the muscle of cultured common carp [22,23], according to Equation (2):

(2) $$BCF=\frac{C_{A}}{C_{B}}\times 100\%$$

where C_A_ is the HCH and DDT content in the muscle of cultured common carp (μg·kg^−1^), and C_B_ is the HCH and DDT content in the sediment (μg·kg^−1^).

### 2.4. Analysis Conditions of Gas Chromatography-Tandem Mass Spectrometry

Separation was performed using a DB-5MS analytical column (30 m length × 0.25 mm I.D; particle size, 0.25 µm). The inlet temperature was 250 °C. The temperature of the oven was maintained at 60 °C for 1 min; it was subsequently increased by 20 °C·min^−1^ to 180 °C and then by 5 °C·min^−1^ to 220 °C, which was maintained for 3 min; finally, it was increased by 25 °C·min^−1^ to 280 °C for 10 min; the total time was 30.4 min. The mass spectrometer parameters were set as follows: electron impact (EI) ionization mode with 70 eV electron energy; transmission line temperature, 280 °C; mass spectrometer ion source temperature, 230 °C; solution delay, 8.0 min; and carrier gas, 1 mL·min^−1^ of helium gas (99.999%). See the literature for specific mass spectrum parameters [20].

### 2.5. Health Risk Assessment Methods

This paper studies human exposure levels resulting from pesticides in the muscle of common carp and sediment. Estimated Daily Intakes (EDI) [24] were analyzed to assess the dietary exposure of residents using guidelines from the Food and Agriculture Organization/World Health Organization (FAO/WHO). The Risk-Based Corrective Action (RBCA) model [25] was utilized to enhance the assessment, incorporating Chronic Daily Intake (CDI) for human health risk assessment. Carcinogenic risk refers to the probability that exposure to OCPs in the human environment might cause cancer. The non-carcinogenic risk was expressed and assessed by the ratio of intake to the reference dose over a given period of time. A Cancer Risk Index (CRI) [24,26] and Hazard Quotient (HQ) were employed to evaluate the health risk associated with population exposure to OCPs. The average daily exposure dose denoting the quantity of pollutants entering the human body through inhalation, ingestion, and dermal contact was individually calculated according to Formulas (3)–(7):

(3) $$EDI=\frac{Cm\times DI\times CF}{BW}$$

(4) $${CDI}_{ingest}=\frac{C_{m}\times {IR}_{ingest}\times EF\times ED\times CF}{BW\times AT\times 1000}$$

(5) $${CDI}_{dermal}=\frac{C_{m}\times {IR}_{dermal}\times EF\times ED\times CF}{BW\times AT\times 1000}$$

(6) $${CDI}_{inhale}=\frac{C_{m}\times {IR}_{inhale}\times EF\times ED}{BW\times AT\times DDF\times 1000}$$

(7) $${IR}_{dermal}=SA\times AF\times ABS$$

where EDI is the daily intake of HCHs or DDTs from the muscle of cultured common carp per person [mg·(kg·d)^−1^]; CDI_ingest_, CDI_dermal_, and CDI_inhale_ are the average daily exposure dose for oral intake, skin contact and respiratory inhalation [mg·(kg·d)^−1^], respectively; and C_m_ is the concentration of HCHs or DDTs in muscle or sediment (μg·kg^−1^). Other parameters are shown in Table 1.

Estimations of CRI [24,26] and HQ (or HI) [27] were based on the Cancer Slope Factor (CSF, kg·d·mg^−1^) and the Reference Dose (RfD, mg·(kg·d)^−1^) of HCHs and DDTs (Table 2) [28,29], as shown in Formulas (8)–(10):

(8) CRI=EDI×CSF

The total carcinogenic risk is the sum of the carcinogenic risk of HCH and DDT monomers.

(9) $$HQ=\frac{EDI}{RfD}$$

If the pollutants are a mixture, the hazard index is HI, as shown in Formula (10):

(10) $$HI=\sum HQi=\sum \frac{{EDI}_{i}}{{RfD}_{i}}$$

The risk is considered acceptable when the risk index is less than 1; the risk is considered unacceptable if the value is greater than 1 [24,30].

### 2.6. Quality Assurance and Quality Control

In this study, the whole process of glass instrument cleaning, sampling, media pretreatment, sample collection, storage and extraction was strictly operated and monitored in accordance with USEPA’s QA/QC requirements. Parallel samples, blank samples, solvent blanks and standard recoveries were included in each batch inspection process. To minimize the impact on test results, one quality control sample was analyzed for every ten samples. The concentration of target compounds in the blank samples was mostly lower than the detection limit. A linear phase relationship between HCHs and DDTs was detected in the range of 0.9958–0.9981; the matrix recovery was 92.53–96.40%, and the relative standard deviations (RSDs) were 0.89–3.85%. The accuracy (recovery rate 70–120%) and precision of the method that were obtained (RSDs < 10%) meet the detection requirements.

### 2.7. Data Statistics and Analysis

The data were expressed as the median value (p 25, p 75) at various time points, with all test samples measured in triplicate. Initial data analysis was conducted using Microsoft office 2010 statistical software, followed by statistical analysis using SPSS 22.0. Visualization of the data was achieved using Microsoft Visio Pro 2016 and Origin Pro 2018 64-bit software.

## 3. Results and Discussion

### 3.1. Residual Levels of HCHs and DDTs

#### 3.1.1. Residual Levels of HCHs and DDTs in the Muscle of Common Carp

**Figure 1:**
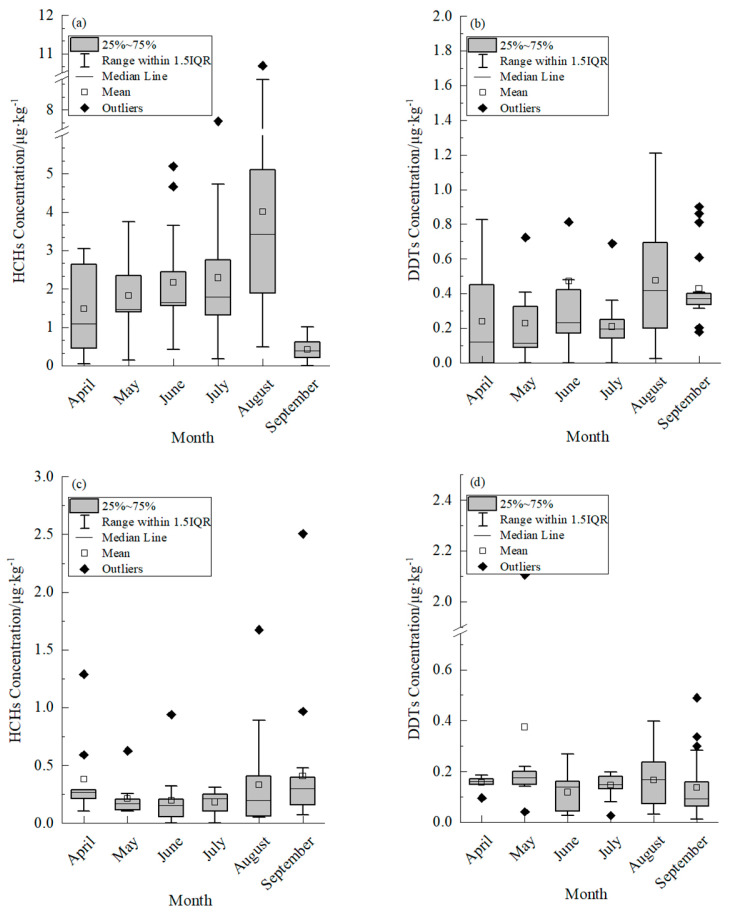
Residual characteristics of Hexachlorocyclohexane (HCHs) and Dichlorodiphenyltrichloroethane (DDTs) in muscle of cultured common carp. and sediment. (**a**) Residual characteristics of HCHs in muscle of cultured common carp. (**b**) Residual characteristics of DDTs in muscle of cultured common carp. (**c**) Residual characteristics of HCHs in sediment. (**d**) Residual characteristics of DDTs in sediment.

The concentration of HCHs in the muscle tissue of cultured common carp exhibited a fluctuating pattern over the course of the seasons, as depicted in Figure 1a. In April, the residual level of HCHs in the muscle was measured at 1.095 μg·kg^−1^ (with a range of 0.410 μg·kg^−1^ to 2.730 μg·kg^−1^), followed by a gradual increase from May to July. The peak residual level and highest enrichment capacity of HCHs in the muscle tissue of cultured common carp was observed in August, with a recorded value of 3.418 μg·kg^−1^ (ranging from 1.832 μg·kg^−1^ to 5.161 μg·kg^−1^) and a BCF of 17.09, as detailed in Table 3. This phenomenon might be attributed to the rainy season in August, during which substantial rainfall resulted in water movement from the adjacent farmland into the breeding pond. The fish accumulated HCHs and DDTs through ingestion, dermal absorption, and other mechanisms. In addition, the common carp specimens were larger, exhibited increased adiposity, and were more prone to bioaccumulate OCPs in August. The concentration of HCHs in the muscle tissue of the collected common carp was below the maximum residue limit for pesticides in food, as specified in the “GB 2763-2021 National Standard for Food Safety [31]”, which sets the maximum residue limit for HCHs at 100 μg·kg^−1^.

The concentration of HCHs in the muscle of cultured common carp was significantly higher compared to *Tachysurus fulvidraco* in the Han River (ranging from 0.18 to 0.89 μg·kg^−1^) [32] and pond-cultured *Siniperca chuatsi* in Wujiang city (ranging from 0.04 μg·kg^−1^ to 1.24 μg·kg^−1^) [33]. The HCH levels in common carp were lower than those found in five species of fish from Taihu Lake (ranging from 0 to 26.8 μg·kg^−1^, fat weight) [34] and the muscle tissue of Squid in the North Pacific squid fishing grounds [35]. The residual levels of HCHs in the muscle tissue of cultured common carp reached their lowest point in September, with a value of 0.387 μg·kg^−1^ (ranging from 0.217 μg·kg^−1^ to 0.642 μg·kg^−1^). There were 1–2 outliers observed in the data, potentially indicating a relationship with fish growth, feeding habits and individual fat content [36].

The levels of DDT residues in the muscle tissue of cultured common carp varied seasonally, as shown in Figure 1b. Specifically, the levels of DDT residues in the muscle of cultured common carp was consistent between April and May, with values of 0.120 μg·kg^−1^ (range: 0, 0.543 μg·kg^−1^) and 0.114 μg·kg^−1^ (range: 0.084 μg·kg^−1^, 0.367 μg·kg^−1^), respectively, while the enrichment capacity remained similar, as indicated in Table 3. The highest residual level of DDTs in the muscle of cultured common carp occurred in August, with a value of 0.420 μg·kg^−1^ (0.176 μg·kg^−1^, 0.699 μg·kg^−1^), while the highest enrichment capacity of DDTs in the muscle of cultured common carp was observed in September (Table 3). The concentration of DDTs in the muscle tissue of common carp specimens was found to be below the maximum residue limit for pesticides in food as specified by the “GB 2763-2021 National Standard for Food Safety”, which sets the maximum allowable limit for DDTs at 500 μg·kg^−1^.

It is worth noting that the highest concentration of DDTs in the muscle of cultured common carp was lower than that of *Tachysurus fulvidraco* in the Han River (12.03 μg·kg^−1^ to 45.75 μg·kg^−1^) [32], the pond-cultured *Siniperca chuatsi* in Wujiang city (1.30 μg·kg^−1^ to 4.57 μg·kg^−1^), as reported by Wang et al. in 2011 [33], five species of fish from Taihu Lake (73.9 μg·kg^−1^ to 643 μg·kg^−1^, fat weight) [34] (Table S1) and the muscle of Squid in North Pacific squid fishing grounds [35]. The concentration of DDTs in the muscle tissue of cultured common carp was found to be lower than that of HCHs. Variability in the residual levels of DDTs was observed in samples collected simultaneously, with the highest outliers occurring in September. The limited enrichment of DDTs in the muscle tissue of cultured common carp could be attributed to the fat-solubility of both HCHs and DDTs. This limitation was particularly pronounced in April when the fish were in a growth stage characterized by reduced food intake and low body fat percentage. The accumulation of HCHs and DDTs in fish was observed to increase during the latter stage of the culture period (August to September), which is likely due to adaptation to the water temperature, heightened food consumption, elevated activity levels and increased lipid content [37].

#### 3.1.2. Residual Levels of HCHs and DDTs in Sediment

The concentration of residual HCHs in sediment exhibited seasonal fluctuations, as shown in Figure 1c. In April, the residual amount of HCHs in sediment was measured at 0.268 μg·kg^−1^ (0.173 μg·kg^−1^, 0.442 μg·kg^−1^). The value decreased to its lowest level in June with a measurement of 0.153 μg·kg^−1^ (0.056 μg·kg^−1^, 0.228 μg·kg^−1^). The residual amounts of HCHs in July and August were higher than those in June by factors of 0.40 and 0.31, respectively. The highest level of residual HCHs was observed in September, with a value of 0.301 μg·kg^−1^ (0.138 μg·kg^−1^, 0.413 μg·kg^−1^). Wang et al. (2023) found that the residual concentration of HCHs in cultivated soil ranged from 5.57 μg·kg^−1^ to 77.83 μg·kg^−1^, a significantly higher range than the results of the present study [16]. Cheng et al. (2021) investigated OCPs pollution in the surface soil of Huixian Wetland and observed an inverse relationship between OCPs content and soil depth [17]. Additionally, factors such as soil organic matter, viscosity and other physical and chemical indexes were found to influence the downward migration of OCPs [38]. The anomalous results of HCH residue levels in months other than July in this study might be attributed to external factors.

The levels of DDT residues in sediment exhibited seasonal fluctuations, as depicted in Figure 1d. The residue level of DDTs in sediment was 0.161 μg·kg^−1^ (0.150 μg·kg^−1^, 0.172 μg·kg^−1^) in April, reaching its peak in May at 0.176 μg·kg^−1^ (0.145 μg·kg^−1^, 0.211 μg·kg^−1^), and fluctuating between May and September. The residual DDT levels in this study were significantly lower than those reported in cultivated soil by Wang et al. (2023) [16], ranging from 1.20 μg·kg^−1^ to 154.58 μg·kg^−1^. The presence of outliers in the DDT residue data from April to September might be attributed to variations in sampling location, depth, and physical and chemical factors.

### 3.2. Composition Characteristics of HCH and DDT Isomers

**Figure 2:**
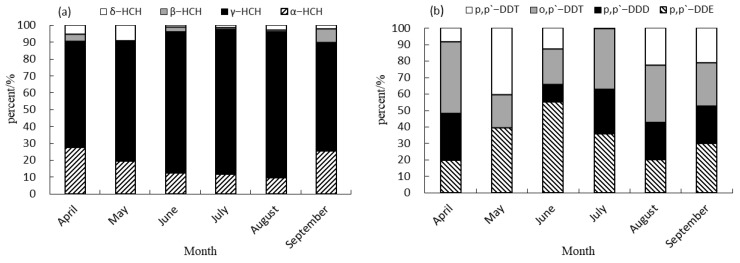
Contribution rate of HCH and DDT isomers in muscle of cultured common carp. (**a**) Contribution rate of HCHs in muscle of cultured common carp. (**b**) Contribution rate of DDTs in muscle of cultured common carp.

The predominant isomers of HCHs and derivatives of DDTs were detected in varying concentrations in the muscle tissue of cultured common carp, as observed in Figure 2. These main components exhibited distinct patterns and varied contribution rates to the overall residue. Specifically, γ-HCH emerged as the primary HCH isomers in common carp muscle samples collected between April and September, with the contribution rate ranging from 62.81% to 86.47%. This prevalence could be attributed to the greater stability of the γ-HCH structure compared to other isomers. It is challenging for indigenous microorganisms in the environment to effectively degrade α-HCH, with β-HCH and δ-HCH contributing only 0 to 9.20% [13]. β-HCH was not detected in the muscle of cultured common carp in May, contrasting with the presence of HCHs in Squid muscles in North Pacific squid fishing grounds as reported by Li et al. (2023) [35]. The primary DDT derivative found in the muscle of cultured common carp was *o*,*p*′-DDT in April, July and August, with a contribution rate ranging from 34.80% to 43.34%. In May, the primary component of the DDT derivatives was identified as *p*,*p*′-DDT, with a contribution rate of 40.42%. Conversely, in June and September, *p*,*p*′-DDE emerged as the predominant component of the DDT derivatives, with contribution rates of 55.29% and 30.02%, respectively. This pattern closely resembled the composition observed in *Squid* muscles within the North Pacific squid fishing ground, where *p*,*p*′-DDE was found in a range from 68.81% to 89.14%) [35].

**Figure 3:**
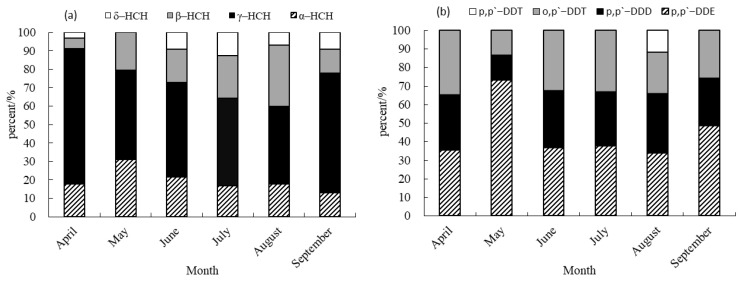
Contribution rate of HCH and DDT isomers in sediment. (**a**) Contribution rate of HCHs in sediment. (**b**) Contribution rate of DDTs in sediment.

The predominant isomers of HCHs and derivatives of DDTs exhibited varying levels of detection in sediment, as depicted in Figure 3. The main components displayed diverse proportions, with different isomers of HCHs and DDT derivatives contributing unequally to the residual quantity. Among the HCH isomers, γ-HCH emerged as the predominant constituent in sediment samples collected from April to September, with a contribution rate ranging from 42.16% to 73.43%. In contrast, δ-HCH exhibited a minimal contribution, ranging from 0 to 12.82%, and was not detected in sediment samples collected in May. The findings of this study align with the results reported by Wang et al. (2023) regarding the prevalence of γ-HCH in cultivated soil [16]. However, the distribution patterns of HCHs in farmland soil of the Jiulong River watershed [15] and the southern Leizhou Peninsula [25] differ from those observed in this study. Specifically, *p*,*p*′-DDE was identified as the primary component of DDTs in sediment samples collected from April to September, with contribution rates ranging from 33.72% to 73.01%. *p*,*p*′-DDT was only detected in August. The contribution rates of *p*,*p*′-DDE and *p*,*p*′-DDD were found to be higher than those of *p*,*p*′-DDT and *o*,*p*′-DDT reported by Liang (2019) [15], who posited that the predominant distribution characteristics of DDTs in farmland soil within the Jiulong River watershed were primarily *o*,*p*′-DDT, a finding which diverges from the results of the present study.

### 3.3. Correlation Analysis of HCHs and DDTs in Muscle of Cultured Common Carp and Sediment

The correlations between HCHs and DDTs in both the muscle tissue of cultured common carp and the sediment are shown in Table 4 and Table 5. A statistically significant positive correlation was observed at a 99% confidence level (*p* < 0.01) between α-HCH and γ-HCH in the muscle of cultured common carp, while a negative correlation at a 99% confidence level (*p* < 0.01) was noted between δ-HCH in the muscle tissue of cultured common carp and α-HCH in sediment. A statistically significant negative correlation was observed between γ-HCH levels in the muscle tissue of cultured common carp and α-HCH levels in the sediment at a confidence level of 99% (*p* < 0.01). A statistically significant negative correlation was found between β-HCH levels in the muscle tissue of cultured common carp and γ-HCH levels in the sediment at a confidence level of 95% (*p* < 0.05), as well as between γ-HCH and δ-HCH in sediment (*p* < 0.05). There was a significant positive correlation between *p*,*p*′-DDD and *o*,*p*′-DDT levels in the muscle tissue of cultured common carp (*p* < 0.01), and between *p*,*p*′-DDD and *o*,*p*′-DDT levels in the sediment *(p* < 0.01).

### 3.4. Health Risk Assessment

The maximum residual levels of HCH isomers or DDT derivatives in the muscle tissue of cultured common carp were considered for the purpose of health risk assessment. The daily fish intake of residents was reported to be 23.7 g·d^−1^ for adults with a body weight (BW) of 60 kg, as per the guidelines set by the Chinese Nutrition Association. The EDI of HCHs (4.292 × 10^−6^ mg·(kg·d)^−1^) was found to be higher than that of DDTs (1.800 × 10^−6^ mg·(kg·d)^−1^). Among the HCH isomers, the EDI was highest for γ-HCH, followed by α-HCH, δ-HCH and β-HCH (Table 6). The EDI of HCHs and DDTs were found to be lower than the RfD, with the CDI ranking in the following order: ingest > dermal > inhale. The CDI resulting from exposure to sediment through various pathways was found to be lower than the EDI from consuming the muscle tissue of cultured common carp, and all CDI values were below the RfD.

Based on the carcinogenic intensity coefficients provided in Table 2, the CRI values of HCHs and DDTs were calculated using Formula (8), with the results presented in Table 7. The CDI ranked in the order of ingest > dermal > inhale. The CRI values of HCHs and DDTs in the muscle tissue of cultured common carp ranged from 1.384 × 10^−7^ to 4.544 × 10^−6^, and from 3.498 × 10^−8^ to 4.029 × 10^−7^, respectively. Consumption of muscle tissue from cultured common carp poses a potential carcinogenic risk due to the maximum CRI of α-HCH and γ-HCH falling between 10^−6^ and 10^−4^. This risk was comparable to that observed in North Pacific squid fishing, with CRI values ranging from 1.29 × 10^−6^ to 5.38 × 10^−6^ [35]. Wang et al. (2011) also suggested that consuming cultured Mandarin fish might present a potential carcinogenic risk [33]. The consumption of grass carp from Baoan Lake might pose potential carcinogenic risks consistent with the findings of this study [39].

The CRI of the sediment via three pathways was found to be lower than that of the muscle tissue of cultured common carp, and was within acceptable limits. The carcinogenic risk of HCHs and DDTs in the sediment was lower than that of soil in agricultural land [25]. The potential carcinogenic risk of HCHs and DDTs in the muscle tissue of cultured common carp was small, and was deemed to be minimal, in the present study.

The HQ values for HCHs and DDTs from ingestion and dermal exposure were found to be equivalent for the population exposed to pond sediment. In the muscle tissue of cultured common carp, the HQ (HI) values for HCHs ranged from 5.02 × 10^−5^ to 1.17 × 10^−2^, while those for DDTs ranged from 3.29 × 10^−4^ to 6.08 × 10^−4^. The non-carcinogenic risk associated with sediment contact in two different pathways was found to be lower than that of consuming the muscle tissue of cultured common carp. The HQ values of HCHs and DDTs were both below 1, indicating a level of risk that was considered acceptable (Table 8). This finding aligns with the conclusions of Wang et al. (2011), who similarly found that the non-carcinogenic risk associated with consuming cultured Mandarin fish posed no significant threat to human health [33]. The public health risk associated with the consumption of signal crayfish polluted by persistent organic pollutants was not significant, as the hazard quotient (HQ) values were below 1 [40]. Liang et al. (2022) observed that the non-carcinogenic risk of HCHs and DDTs in pond sediment was lower for human populations compared with the risk from agricultural soil [25]. According to the results in this study, the carcinogenic risk and non-carcinogenic risk was not significant when consumption of common carp with the highest levels of HCHs did not exceed 2197 g and 12,845 g per week.

## 4. Conclusions

The concentrations of residual HCHs and DDTs in the muscle tissue of common carp were lower than the values set in China’s national limit standard. HCH levels were observed to be higher than DDT levels, with sediment showing lower residual levels compared to the muscle of cultured common carp. The muscle tissue of cultured common carp exhibited a moderate capacity for enrichment of HCHs and DDT_S_. The predominant residual substance of HCHs in both the muscle tissue and sediment of cultured common carp was γ-HCHs, with α-HCH showing significant correlations with γ-HCH and δ-HCH in the muscle tissue, as well as with α-HCH in the sediment (*p* < 0.01). There was a significant negative correlation between γ-HCH and δ-HCH in sediment and β-HCH in muscle of cultured common carp (*p* < 0.05). The main residues of DDT derivatives did not exhibit a clear pattern. The consumption of muscle tissue from cultured common carp poses potential carcinogenic risks associated with α-HCH and γ-HCH. However, the overall carcinogenic and non-carcinogenic risks of OCPs resulting from such consumption were deemed acceptable, with the exception of α-HCH and γ-HCH.

## Figures and Tables

**Table 1:** Parameters in average daily dose calculation models of health risk assessment.

Parameters	Name	Unit	Value
DI	Daily intake of fish by residents	g·d^−1^	23.7
BW	Body weight	kg	60
IR_inhale_	Pollutants inhaled into the human body through respiration	m^3^·d^−1^	15.7
IR_ingest_	Oral ingestion	mg·d^−1^	100
EF	Exposure frequency	d·a^−1^	250
ED	Exposure duration	a	30
AT (carcinogenic)	Average exposure time of contaminants	d	10,950
AT (non-carcinogenic)	Average exposure time of contaminants	d	25,550
CF	Conversion factor	mg ng^−1^;	10^−6^
DDF	Dust diffusion factor of soil	m^3^ kg^−1^	1.316 × 10^9^
SA	The amount of skin area that may be in contact with the soil	cm^2^	5700
AF	Adsorption factor of soil to skin	mg·cm^−2^	0.07
ABS	Skin absorption coefficient	/	0.1

**Table 2:** The cancer slope factor and reference doses of Hexachlorocyclohexane (HCHs) and Dichlorodiphenyltrichloroethane (DDTs) resulting from ingestion of fish and contact with sediment.

OCPs	CSF/kg·d·mg^−1^				RfD/mg·(kg·d)^−1^			
	Ingestion	Oral Exposure	Skin Exposure	Respiratory Intake	Ingestion	Oral Exposure	Skin Exposure	Respiratory Intake
α-HCH	6.3	6.3	6.3	1.8	8 × 10^−3^	8 × 10^−3^	8 × 10^−3^	/
γ-HCH	1.3	1.8	1.8	0.53	3 × 10^−4^	3 × 10^−4^	3 × 10^−4^	/
β-HCH	1.8	1.1	1.1	0.31	5 × 10^−4^	3 × 10^−4^	3 × 10^−4^	/
δ-HCH	1.8	/	/	/	3 × 10^−4^	/	/	/
*p*,*p*′-DDE	0.34	0.34	0.34	0.097	/	2 × 10^−3^	2 × 10^−3^	/
*p*,*p*′-DDD	0.24	0.24	0.24	0.067	/	/	/	/
*o*,*p*′-DDT	0.34	/	/	/	5 × 10^−4^	/	/	/
*p*,*p*′-DDT	0.34	0.34	0.34	0.097	5 × 10^−4^	5 × 10^−4^	5 × 10^−4^	/

Note: “/” indicates no data, the same as below.

**Table 3:** Bioconcentration factors of HCHs and DDTs in muscle of cultured common carp.

OCPs	April	May	June	July	August	September
HCH	4.08	8.74	10.72	8.50	17.09	1.29
DDT	0.75	0.65	1.67	1.31	2.48	4.00

**Table 4:** Correlation analysis of HCH isomer levels in muscle of cultured common carp and sediment.

Pearson		Muscle of Cultured Common Carp				Sediment			
		α-HCH	γ-HCH	β-HCH	δ-HCH	α-HCH	γ-HCH	β-HCH	δ-HCH
Muscle of Cultured common carp	α-HCH	1							
	γ-HCH	0.343 **	1						
	β-HCH	−0.094	0.035	1					
	δ-HCH	−0.307 **	−0.119	−0.179	1				
Sediment	α-HCH	−0.331 **	−0.342 **	0.049	0.122	1			
	γ-HCH	0.062	−0.135	−0.232 *	−0.094	0.037	1		
	β-HCH	0.064	−0.037	−0.123	−0.060	−0.020	0.191	1	
	δ-HCH	−0.070	0.122	−0.072	−0.066	−0.141	−0.235 *	0.084	1

Note: *. At level 0.05 (double-tailed), the correlation was significant; **. At level 0.01 (double-tailed), the correlation was significant.

**Table 5:** Correlation analysis of DDT derivatives in muscle of cultured common carp and in sediment.

Pearson		Muscle of Cultured Common Carp				Sediment			
		*p*,*p*′-DDE	*p*,*p*′-DDD	*o*,*p*′-DDT	*p*,*p*′-DDT	*p*,*p*′-DDE	*p*,*p*′-DDD	*o*,*p*′-DDT	*p*,*p*′-DDT
Muscle of Cultured common carp	*p*,*p*′-DDE	1							
	*p*,*p*′-DDD	0.142	1						
	*o*,*p*′-DDT	0.097	0.415 **	1					
	*p*,*p*′-DDT	−0.026	0.043	−0.165	1				
Sediment	*p*,*p*′-DDE	0.115	0.139	0.002	0.116	1			
	*p*,*p*′-DDD	−0.031	−0.100	−0.052	−0.078	−0.074	1		
	*o*,*p*′-DDT	−0.057	−0.062	−0.015	−0.158	0.008	0.904 **	1	
	*p*,*p*′-DDT	a	a	a	a	a	a	a	a

Note: **—at level 0.01 (double-tailed), the correlation is significant; a—because at least one variable is constant, the correlation cannot be calculated.

**Table 6:** Estimated daily intake of HCHs and DDTs from eating common carp and contact with sediment [mg·(kg·d)^−1^].

OCPs	Muscle of Cultured Common Carp	Sediment		
	EDI	CDI_ingest_ (Non-Carcinogenic/ Carcinogenic)	CDI_dermal_ (Non-Carcinogenic/Carcinogenic)	CDI_inhale_ (Non-Carcinogenic/ Carcinogenic)
α-HCH	0.401 × 10^−6^	1.801 × 10^−10^/7.720 × 10^−11^	7.187 × 10^−11^/3.080 × 10^−11^	2.149 × 10^−14^/ 9.210 × 10^−15^
γ-HCH	3.495 × 10^−6^	2.495 × 10^−9^/ 1.069 × 10^−9^	9.953 × 10^−10^/4.266 × 10^−10^	2.976 × 10^−13^/ 1.275 × 10^−13^
β-HCH	0.077 × 10^−6^	2.614 × 10^−10^/1.120 × 10^−10^	1.043 × 10^−10^/4.470 × 10^−11^	3.119 × 10^−14^/ 1.337 × 10^−14^
δ-HCH	0.319 × 10^−6^	1.554 × 10^−10^/6.659 × 10^−11^	6.199 × 10^−11^/2.657 × 10^−11^	1.854 × 10^−14^/ 7.944 × 10^−15^
*p*,*p*′-DDE	1.185 × 10^−6^	2.283 × 10^−9^/ 9.785 × 10^−10^	9.110 × 10^−10^/ 3.904 × 10^−10^	2.724 × 10^−13^/ 1.167 × 10^−13^
*p*,*p*′-DDD	0.146 × 10^−6^	2.140 × 10^−10^/9.173 × 10^−11^	8.540 × 10^−11^/3.660 × 10^−11^	2.554 × 10^−14^/ 1.094 × 10^−14^
*o*,*p*′-DDT	0.165 × 10^−6^	2.156 × 10^−10^/9.242 × 10^−11^	8.604 × 10^−11^/3.687 × 10^−11^	2.573 × 10^−14^/ 1.103 × 10^−14^
*p*,*p*′-DDT	0.304 × 10^−6^	0/0	0/0	0/0
HCHs	4.292 × 10^−6^	3.091 × 10^−9^/ 1.325 × 10^−9^	1.233 × 10^−9^/ 5.286 × 10^−10^	3.688 × 10^−13^/ 1.581 × 10^−13^
DDTs	1.800 × 10^−6^	2.713 × 10^−9^/ 1.163 × 10^−9^	1.082 × 10^−9^/ 4.639 × 10^−10^	3.236 × 10^−13^/ 1.387 × 10^−13^

**Table 7:** Carcinogenic risk index of HCHs and DDTs.

OCPs	Muscle of Cultured Common Carp	Sediment		
	CRI	CRI_ingest_	CRI_dermal_	CRI_inhale_
α-HCH	2.528 × 10^−6^	4.864 × 10^−10^	1.941 × 10^−10^	1.658 × 10^−14^
γ-HCH	4.544 × 10^−6^	1.924 × 10^−9^	7.678 × 10^−10^	6.760 × 10^−14^
β-HCH	1.384 × 10^−7^	1.232 × 10^−10^	4.917 × 10^−11^	4.143 × 10^−15^
δ-HCH	5.736 × 10^−7^	/	/	/
*p*,*p*′-DDE	4.029 × 10^−7^	3.327 × 10^−10^	1.327 × 10^−10^	1.132 × 10^−14^
*p*,*p*′-DDD	3.498 × 10^−8^	2.202 × 10^−11^	8.784 × 10^−12^	7.332 × 10^−16^
*o*,*p*′-DDT	5.594 × 10^−8^	/	/	/
*p*,*p*′-DDT	1.034 × 10^−7^	0	0	0

**Table 8:** Non-carcinogenic risk index of HCHs and DDTs.

OCPs	Muscle of Cultured Common Carp	Sediment		
	HQ	HQ_ingest_	HQ_dermal_	HQ_inhale_
α-HCH	5.02 × 10^−5^	2.252 × 10^−8^	2.252 × 10^−8^	/
γ-HCH	1.17 × 10^−2^	8.315 × 10^−6^	8.315 × 10^−6^	/
β-HCH	1.54 × 10^−4^	8.714 × 10^−7^	8.714 × 10^−7^	/
δ-HCH	1.06 × 10^−3^	/	/	/
*p*,*p*′-DDE	/	1.142 × 10^−6^	1.142 × 10^−6^	/
*p*,*p*′-DDD	/	/	/	/
*o*,*p*′-DDT	3.29 × 10^−4^	/	/	/
*p*,*p*′-DDT	6.08 × 10^−4^	0	0	/

## Data Availability

The original contributions presented in this study are included in the article. Further inquiries can be directed to the corresponding authors.

## Supplementary Materials

The following supporting information can be downloaded at: https://www.mdpi.com/article/10.3390/foods14020223/s1, Table S1: Comparison of HCHs and DDTs content in common carp muscle with other studies (μg·kg^−1^).
