# Supplementary material for: Residual, Enrichment and Health Risk Assessment of Hexachlorocyclohexane and Dichlorodiphenyltrichloroethane in Muscle of Cultured Common Carp
Source: Foods. 2025 Jan 13;14(2):223. doi: 10.3390/foods14020223 (PMC11765017; doi:10.3390/foods14020223)
Supplement: Supplementary file 1 [file foods-14-00223-s001.zip › foods-3371454-supplementary.pdf]

Residual, Enrichment and Health Risk Assessment of Hexachlorocyclohexane and Dichlorodiphenyltrichloroethane in Muscle of Cultured Common Carp

Li Huang,[a,b] Lei Gao,[a,b] Song Wu,[a,b] Peng Wang,[a,b] Zhongxiang Chen,[a,b] Qirui Hao,[a,b] Dongli Qin,[a,b,*] and Xiaoli Huang[a,c,*]

a. Heilongjiang River Fisheries Research Institute, Chinese Academy of Fishery Sciences, Harbin, Heilongjiang, China
b. Inspection and Testing Center for Fishery Environment and Aquatic Products (Harbin), Ministry of Agriculture and Rural Affairs, Harbin, Heilongjiang, China
c. Heilongjiang River Basin Fisheries Ecology Observation and Research Station of Heilongjiang Province, Harbin, Heilongjiang, China
* Correspondence: Correspondence: huangxiaoli@hrfri.ac.cn (Xiaoli Huang); qindongli@hrfri.ac.cn (Dongli Qin)

Table S1: Comparison of HCHs and DDTs content in common carp muscle with other studies ($\mu g \cdot kg^{-1}$)

Area	Research object	HCHs	DDTs	Reference
Harbin City	common carp	3.418	0.420	This research
Han River	*Tachysurus fulvidraco*	0.18 - 0.89	12.03 - 45.75	(Zhang et al., 2014)
Wujiang city	*Siniperca chuatsi*	0.04 - 1.24	1.30 - 4.57	(Wang et al., 2011)
Taihu Lake	Five species of fish	0 - 26.8	73.9 - 643	(Li, 2019)
